# Supplementary material for: The Validity and Feasibility of Utilizing the Photo-Assisted Dietary Intake Assessment among College Students and Elderly Individuals in China
Source: Nutrients. 2024 Jan 9;16(2):211. doi: 10.3390/nu16020211 (PMC10818835; doi:10.3390/nu16020211)
Supplement: Supplementary file 1 [file nutrients-16-00211-s001.zip › nutrients-2741942-supplementary.pdf]

nutrients

MDPI

Supplementary Materials

Method

Design and production of the food atlas

The food atlas, specially developed to assess the mass by conversing food volume in the special designed bowls into food mass, contained 150 images of 70 food types. Each food is presented in two images from two different angles (directly above at a 45 °angle in front, and at a 45 ° angle side), and each image shows foods with half or one unit volume of bowls (i.t. 60 or 120 cm^3).

Basically, the bowls designed as a series of five bowls with the same base area (120cm2) and different heights showing different functions，for holding staple foods (height of 5 cm), vegetables (height of 3 cm), meats (height of 2 cm), fruits or nuts (height of 2 cm)(Fig.1).

The core function for food atlas set up in five steps,

(1) Collection of food item including processing and cooking methods

We took photo more than 45 types dishes based on the ordinary food item including some food with Chinese characteristics, such as Yu-Shiang shredded pork，hot-spicy pot, blood curd, etc., all of which were displayed in detail. Meanwhile, the same food with different cooking was also considered. For example, Cold eggplant is steamed, and a lot of water is lost during steaming, so the ratio of raw/cooked is usually 50%, while roasted eggplant is deep-fried and coated with a batter (e.g., flour, egg wash) prior to deep-frying, so that not only is the loss of water in the eggplant after frying very low, but the weight of the eggplant is increased.

(2) Determine the percentage of edible portion for foods and raw/cooked ratio,

For cooking each dish, each food was cooked separately and then mixed into dishes. Before and after cooking, the raw and cooked mass of each food were accurately weighed, and the corresponding raw/cooked ratio was calculated, and If there were still some inedible parts, such as bones after cooking, the 100% edible weight of each kind of food was calculated according to the proportion of edible parts provided by the Chinese Food Composition Tables (6th edition). Therefore, the atlas could provide the percentage of edible parts, raw/cooked ratio, dry/wet ratio and other information

(3) Determination of sizing reference and portion sizes

The cooked foods were served into a clean specially designed bowls with half or one unit volume of bowls (i.t. 60 or 120 $cm^{3,}$ which was calculated from one/half base area of 120 cm^2 or 60 cm^2 centimeters and a unit height（1cm). It is important that the food was put on the same level and without the juice of the dish.

(4) Photographic records of food pictures

The food was photographed from two angles: directly above, at a 45° angle in front. Then, according to the result of the previous weighing, the food weight information of the unit portions was determined.

(5) Food pictures collection to form food atlas

The pictures of all types of food blended with the common cooking and state of Chinese homemade dishes, which were summarized and classified into 13categories, including 1) cereals and its products; 2)turber; 3) vegetables; 4) fruits; 5) livestock meat including organs and blood products ;6) poultry meat including organs and blood products; 7) fish, shrimp and shellfish; 8) eggs; 9) soybeans and its products; 10) nuts; 11) mushrooms and algae; 12) cakes, candies and condiments; 13)beverages; and 14) starch products.

Then, they were combined with the relevant food information, such as the percentage of edible parts, raw/cooked weight ratio and dry/wet weight ratio. Finally, the information

about unit volume and its corresponding mass were determined for food atlas to be used for dietary assessment

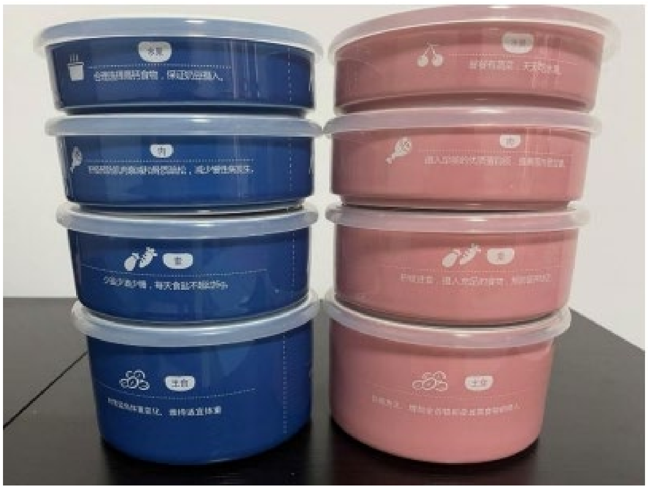

Figure S1. The special designed bowls for PAD.

Table S1. The participants information.

	**College students(n=76)**	**Elders(n=121)**
Age(years)	22.37±1.03	65.65±2.59
Gender		
Male	48(63.16)	40(33.06)
Female	28(36.84)	82(67.77)
Nationality		
Han	74(97.37)	120(99.17)
other	2(2.63)	1(0.83)
Education level		
Primary school and below	0(0)	5(4.13)
Junior high school	0(0)	45(3.31)
High school/technical school	76(100)	42(34.71)
University and above	0(0)	30(24.79)

Table S2. The menu and food items in the validation of PAD.

**Cereals**	**Vegetarian dish**	**half-vegetarian dish**	**meats**	**eggs**	**Tubers**
rice	Saute cabbage	Saute shredded pork with eggs &black fungus	Pork fillets with sweet & sour sauce	steamed egg custard	steamed sweet potato
steamed buns	• Stir-fried bean sprouts	Fried pork with peppers	Steamed fish fillets	Saute leek sprouts &eggs	Steamed Yam
fried rice with egg	Mapo beancurd	Kung Pao chicken	Steamed spare ribs	Scrembled Eggs with tomato	fried patato
fried noodles	Braised green bean	Yu-shiang shredded pork	Boiled beef slices		spicy and sour shredded potato
	• Stir-fried mushrooms with rape		Stir-fried sliced lamb with scallion		
	• Stir-fried winter melon		Braised pork balls		

	• Stir-fried lettuce<br>Eggplant with Chilli and Potato<br>Braised vermicelli with cabbage	
